# Subtractive Low-Temperature Preparation Route for Porous SiO_2_ Used for the Catalyst-Assisted Growth of ZnO Field Emitters

**DOI:** 10.3390/nano11123357

**Published:** 2021-12-10

**Authors:** Stefanie Haugg, Carina Hedrich, Robert H. Blick, Robert Zierold

**Affiliations:** 1 Center for Hybrid Nanostructures (CHyN), Universität Hamburg, 22761 Hamburg, Germany; shaugg@physnet.uni-hamburg.de (S.H.); chedrich@physnet.uni-hamburg.de (C.H.); rblick@physnet.uni-hamburg.de (R.H.B.); 2 Material Science and Engineering, College of Engineering, University of Wisconsin-Madison, Madison, WI 53706, USA

**Keywords:** porous SiO_2_, ZnO nanowires, membranes, field emission, MOCVD

## Abstract

The possibility to gradually increase the porosity of thin films facilitates a variety of applications, such as anti-reflective coatings, diffusion membranes, and the herein investigated tailored nanostructuring of a substrate for subsequent self-assembly processes. A low-temperature (<160 °C) preparation route for porous silicon oxide (porSiO_2_) thin films with porosities of about 60% and effective refractive indices down to 1.20 is tailored for bulk as well as free-standing membranes. Subsequently, both substrate types are successfully employed for the catalyst-assisted growth of nanowire-like zinc oxide (ZnO) field emitters by metal organic chemical vapor deposition. ZnO nanowires can be grown with a large aspect ratio and exhibit a good thermal and chemical stability, which makes them excellent candidates for field emitter arrays. We present a method that allows for the direct synthesis of nanowire-like ZnO field emitters on free-standing membranes using a porSiO_2_ template. Besides the application of porSiO_2_ for the catalyst-assisted growth of nanostructures and their use as field emission devices, the herein presented general synthesis route for the preparation of low refractive index films on other than bulk substrates—such as on free-standing, ultra-thin membranes—may pave the way for the employment of porSiO_2_ in micro-electro-mechanical systems.

## 1. Introduction

Porous silicon oxide (porSiO_2_) is often investigated as an anti-reflective coating (ARC) since the low-cost material can be generated by a multitude of techniques and its refractive index (RI) can be tailored over a wide range below 1.46, which is the typical RI of dense SiO_2_ [1,2,3,4,5]. Note, a material in solid form with an RI lower than that of magnesium fluoride (1.38) is not available in nature but can technically be overcome by the artificial introduction of pores in a thin film [4].

Besides the application of porSiO_2_ as an ARC for photovoltaics, it is also used for the reduction of reflection from optical equipment, such as camera lenses and eyeglasses, or for the fabrication of anti-glare displays [2,6,7]. Moreover, porSiO_2_ is proposed as a diffusion membrane for the separation and purification of gases or liquids as well as for the protective encapsulation of nanostructures. Porous films are also reported as thermal insulation layers in uncooled infrared detectors and as biocompatible substrates because their roughness can enhance the growth and proliferation of cells [8,9,10]. A frequently used synthesis method for porSiO_2_ is the simple sol-gel technique. However, it requires a calcination step at temperatures well above 150 °C for the complete removal of the solvents, which provokes cracking of the remaining porous layer and prohibits the coating of temperature-sensitive substrates. Additionally, the adjustment of the RI and of the film thickness is known to be challenging, and this technique often leads to inhomogeneous deposits on non-planar surfaces [2,3,11]. Therefore, plasma-enhanced chemical vapor deposition (PECVD) was introduced as a dry coating method that permits the direct synthesis of porous films without the need for post-deposition steps, such as annealing. By extreme fine-tuning of the deposition recipe, SiO_2_ particles can be directly created in the gas phase and are subsequently accumulated as a porous layer on the substrate [3,9]. However, the generated films of aggregated particles typically suffer from poor mechanical stability, which is a critical property for many applications, such as outdoor ARCs [5,10].

Herein, we present a subtractive preparation route for porSiO_2_ thin films, which involves the deposition of silicon oxynitride (SiO_x_N_y_) films by PECVD, followed by the selective removal of the silicon nitride (SiN_y_) compounds by chemical wet etching in heated phosphoric acid (H_3_PO_4_). The as-deposited films, as well as the porous layers, are investigated by spectral ellipsometry to analyze the RI reduction and to determine the apparent porosity of the remaining film. Specifically, the RI of the starting material SiO_x_N_y_ is tailor-made set to exactly control the amount of incorporated SiN_y_ compounds by adjustment of the gas flow ratios in the PECVD process. Consequently, the porosity after wet chemical etching, and, thus, the RI of the porSiO_2_, can be finely tuned and lead to a minimum RI of 1.2 at 632.8 nm, which corresponds to a porosity of about 60%. For the preparation on free-standing SiN_y_ membranes, modifications of the synthesis route for porSiO_2_ on bulk silicon (Si) substrates had to be established. Both substrate types, namely porSiO_2_ on bulk and free-standing membranes, are employed as bases for the catalyst-assisted growth of zinc oxide (ZnO) nanowires (NWs) by metal organic chemical vapor deposition (MOCVD). The porSiO_2_ is found to facilitate the formation of catalyst particles from a thin gold film by thermally induced solid-state dewetting well below the bulk material’s melting point [12]. Note, the electron field emission (FE) properties of an emitter are mainly determined by its electronic surface structure and its geometrical shape. The latter defines the intensity of the geometrical field enhancement effect and, therefore, strongly affects the electric field needed to allow for electron emission by FE. ZnO nanostructures are well-known for their excellent FE performance because they can be grown with large aspect ratios (ratio of length to diameter), especially when their synthesis is combined with a catalyst [13,14]. Moreover, the transparent II-VI compound semiconductor exhibits good thermal and chemical stability, which permits its application in harsh environments [15,16]. If a field emitter array were placed on a flexible membrane, it could potentially act as an FE-based pressure sensor or as a detector for mass spectrometry of heavy biomolecules. Principally, its function relies on the recognition of the FE current change, which corresponds to the magnitude of the membrane displacement by the external force [17,18,19]. The herein investigated ZnO NWs on free-standing ultra-thin membranes prepared with the help of the porSiO_2_ may have the potential to be used in future sensor applications because of their reproducible FE properties combined with the sufficient stability of the emission current in the microampere range over several hours.

## 2. Materials and Methods

### 2.1. Preparation Route for Porous SiO_2_

The dielectric films were deposited by PECVD (SI 500 D, Sentech Instruments GmbH, Berlin, Germany) at a temperature of 130 °C using the following parameters: 3 Pa chamber pressure, 450 W power, 145 sccm of 5% silane (SiH_4_, purity 5.0) in helium (He, purity 5.6), and 126 sccm argon (Ar, purity 6.0). For the deposition of SiO_2_ films, oxygen flows (O_2_, purity 5.0) between 15 sccm and 35 sccm were used, and, for the SiO_x_N_y_ films, 15 sccm up to 30 sccm ammonia (NH_3_) were added to the recipe with a fixed O_2_ flow of 30 sccm.

For the synthesis of porSiO_2_ on Si bulk substrates, extra pure 85% H_3_PO_4_ (100 mL, Carl Roth) was heated for 30 min in a round bottom flask in a heating mantle before the sample, which was clamped in a tailor-made PTFE holder, was lowered into the boiling hot 154 °C etchant. A thermometer, a reflux condenser, and the sample holder were assembled on the three necks of the flask. The sample was rinsed in ultrapure water (water purification system Milli-Q^®^ Integral 5, Merck, Darmstadt, Germany) to terminate the chemical reaction after an etch duration of 30 min, then it was soaked in ethanol, and, finally, it was dried with gaseous nitrogen. However, for the synthesis of porSiO_2_ on free-standing membranes, the sample was placed on a tailor-made PTFE holder without clamping to avoid any mechanical stress applied to the fragile substrate, and the sample was heated within the H_3_PO_4_ (30 mL) on a hot plate to circumvent sudden temperature changes. The etching was initialized by setting the cold hot plate to 150 °C, and a glass plate was loosely placed on the beaker to prevent strong concentration changes by water evaporation. Note, the same volume of liquid was used for each fabrication run to allow for a reproducible etch process. However, the temperature of the heated H_3_PO_4_ was not measured to avoid water evaporation when lifting the glass plate. After the etch time passed, the sample was transferred to heated ultrapure water to terminate the chemical reaction, and then it was rinsed in methanol. Finally, the sample drying was accelerated on the hot plate, which was still set to 150 °C to prevent the gathering of solvents and the subsequent formation of residues on the flexible membrane part of the sample. The film thicknesses and RIs were measured by spectroscopic ellipsometry (SENpro, Sentech Instruments GmbH, Berlin, Germany) before and after chemical wet etching as an indication for the composition of the as-deposited films and to estimate the apparent porosity from the RI reduction, respectively. Note, all measured RIs are given for a wavelength of 632.8 nm.

### 2.2. Synthesis of Nanowire-like ZnO Field Emitters

The catalyst-assisted MOCVD of ZnO NWs was carried out in a horizontal three-zone tube furnace (OTF-1200X-III-UL, MTI corporation, Richmond, CA, USA) with the precursors zinc acetylacetonate hydrate powder (Zn(acac)_2_, from Sigma-Aldrich, 2.4 g, Taufkirchen, Germany) and O_2_ (100 sccm, purity 5.0), as well as with Ar (83 sccm, purity 5.0) as a transport gas. A detailed description of the growth process execution is given in our previous work about the catalyst-free growth of ZnO nanowhiskers [20]. Note, a growth temperature of 580 °C and a growth time of 12 h were applied in the present study. Thin gold films were sputtered with a sputter coater (K550X, Emitech, Ashford, Kent, England) on the porous SiO_2_ films on bulk and on free-standing membranes prior to the ZnO growth process. No intermediate annealing step was needed since the thermally induced catalyst particle formation took place during the heating ramp of the ZnO growth process. The SEM images of the synthesized structures were evaluated using ImageJ [21] to extract the mean particle diameter of the gold particle distribution after dewetting (threshold and analyze particles functions) and to approximate the mean emitter dimensions by analysis of 20 large ZnO NWs. The NW lengths measured in the SEM images were divided by the cosine of the stage tilt (54°) to derive corrected estimates because tilting of the SEM stage is known to lead to a distortion of imaged objects that are oriented perpendicular to the tilt axis [22].

### 2.3. Field Emission Measurements

The FE measurements were executed in two in-house built FE setups using a triode-type electrode arrangement. An electric field applied between the emitter sample and a nickel grid (59 lines per centimeter, Precision Eforming) was ramped while the current was measured at a polished metal anode plate. A constant voltage of −100 V was supplied to the grid electrode, and the absolute value of the negative voltage applied to the emitter sample was increased stepwise using two separate high voltage power supplies (PS350, Stanford Research Systems, Sunnyvale, CA, USA). Emitter and grid electrode were electrically insulated from each other by a 250 µm thick PTFE sheet with a centered square hole that exposed a macroscopic emission area of 0.16 cm^2^. In the first FE setup (base pressure 2 × 10^−4^ Pa), a transimpedance current amplifier (DLPCA-200, Femto Messtechnik GmbH, Berlin, Germany) was used for signal amplification. The signal was integrated over a large number of data points (2 × 10^6^ samples, 10^6^ samples/s) to obtain a single current reading in order to suppress statistical error sources [23]. In the second FE setup (base pressure 1 × 10^−5^ Pa), the FE current was directly measured with a picoammeter (model 6485, Keithley, Cleveland, OH, USA). The offsets and the linear slopes of the measured current–voltage curves caused by the inherent offsets of the measurement devices and by the leakage current in the measurement assembly, respectively, were subtracted prior to further data analysis [23]. All measurements were carried out at least 72 h after mounting an as-deposited emitter sample in vacuum to allow for proper outgassing, which showed to enhance the reproducibility of the measured current–voltage curves and lowered the risk for the occurrence of an electrical breakdown.

## 3. Results

### 3.1. Synthesis of Porous SiO_2_ on Bulk Substrates

**Figure 1:**
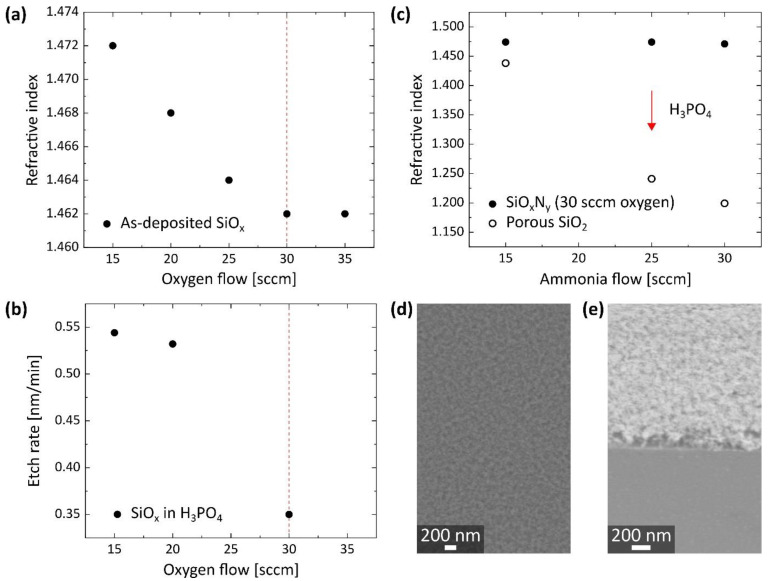
Synthesis of porSiO_2_ by selective wet etching of PECVD SiO_x_N_y_. (**a**) The RI of PECVD SiO_x_ decreased gradually with increasing oxygen flow and reached a minimum of 1.462 for 30 sccm. (**b**) The PECVD SiO_x_ deposited with 30 sccm oxygen exhibited the strongest resistance to boiling H_3_PO_4_. (**c**) The RI reduction by selective wet etching of SiO_x_N_y_ in boiling H_3_PO_4_ was enhanced with increasing ammonia flow in the PECVD recipe. The lowest effective RI of porSiO_2_ was achieved by wet etching of SiO_x_N_y_ that was deposited with 30 sccm oxygen and 30 sccm ammonia. (**d**) Top and (**e**) side view SEM images of porSiO_2_ with an effective RI of 1.206 and a thickness of 52 nm. The SEM images were taken with the Supra 55 by Zeiss and the side view was taken at an angle of 30°.

The subtractive synthesis route for porSiO_2_ on bulk Si includes the following main steps: first, the deposition of SiO_x_N_y_ by PECVD, and, second, the selective removal of the SiN_y_ compounds by wet etching in boiling H_3_PO_4_, which is refluxed to maintain a stable etch rate [24]. Initially, the SiO_x_ PECVD recipe was optimized to obtain an RI close to its typical value of 1.46 [3,25]. As shown in Figure 1a, the RI of the SiO_x_ decreased gradually with increasing O_2_ gas flow and a minimum of 1.462 was reached for 30 sccm close to the RI values of bulk and of atomic layer deposited SiO_2_ [26]. Moreover, the silicon oxide deposited with 30 sccm O_2_ showed the strongest resistance to the boiling H_3_PO_4_ (Figure 1b), which is, from here on, referred to as SiO_2_. Subsequently, NH_3_ was added to the SiO_2_ recipe to generate SiO_x_N_y_ films. Since the RI of the as-deposited SiO_x_N_y_ varied only in a narrow range around 1.473 ± 0.002 as a function of the NH_3_ content (Figure 1c, filled circles), the Si-O bond formation appears to have been the dominant mechanism [27]. Moreover, the growth rate increased from 19.0 nm/min to 21.7 nm/min with rising NH_3_ flow. The effective RI obtained after etching in H_3_PO_4_ decreased from 1.44 to 1.20 with rising NH_3_, as displayed in Figure 1c (empty circles). As expected, a larger amount of SiN_y_ compounds initially incorporated into the SiO_x_N_y_ film—corresponding to an increased NH_3_ flow—led to a larger RI reduction and, potentially, to an enhanced porosity of the remaining SiO_2_. However, further RI reduction by extended etching was not possible since it caused a strong decrease in the film thickness (from 87 nm as-deposited to below 10 nm after etching) along with an increase in the RI above the initial value of the as-deposited SiO_x_N_y_. This observation could suggest the total collapse of the porous structure. In Figure 1d,e, the structural constitution of porSiO_2_ is shown, which was generated from a SiO_x_N_y_ film deposited with 30 sccm NH_3_ and 30 sccm O_2_, which is, from here on, exclusively used as the PECVD recipe in this study.

### 3.2. Synthesis of Porous SiO_2_ on Free-Standing Membranes

**Figure 2:**
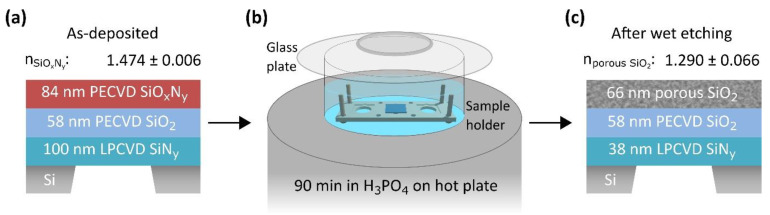
Process transfer of the porSiO_2_ synthesis from bulk substrates to free-standing membranes. (**a**) In the first step, a LPCVD SiN_y_ membrane supported by a Si substrate was covered with SiO_2_ and subsequently with SiO_x_N_y_ by PECVD. (**b**) In the second step, the sample was placed on a tailor-made PTFE sample holder in aqueous H_3_PO_4_ on a hot plate, which was set to 150 °C, to selectively remove the SiN_y_ compounds from the SiO_x_N_y_. A glass plate was loosely assembled on the beaker to avoid concentration changes in the etchant by water evaporation. (**c**) The effective RI of the porSiO_2_ was repeatedly reduced to 1.290 by etching of the sample for 90 min. Simultaneously, ultra-thin membranes were generated by thickness reduction of the lowest LPCVD SiN_y_ layer in the H_3_PO_4_. The etchant had access to the membrane’s backside through the hole in the sample holder, which allowed for the SiN_y_ thickness reduction with a rate of (0.65 ± 0.04) nm/min.

The previously applied synthesis route for porSiO_2_ on bulk substrates strongly decreased the yield of intact SiN_y_ membranes when the same synthesis approach was used. Most likely, the etching in the boiling H_3_PO_4_ was too harsh for the free-standing membranes or they were destroyed by the sudden temperature and ambient condition changes when transferred from one solution to another. As a consequence, adjustments were needed to allow for the synthesis of porSiO_2_ on 100 nm thick 2.6 × 2.6 cm^2^ SiN_y_ membranes, which were fabricated in-house according to the conventional sequence of photolithography and etching from commercially bought Si wafers coated with low-pressure chemical vapor deposited (LPCVD) SiN_y_ on both sides [28]. Firstly, a thin layer of SiO_2_ was deposited on the SiN_y_ membrane before the SiO_x_N_y_ was generated to increase the substrate’s durability for the subsequent wet etching. Both thin films were successively deposited for 4 min each by PECVD without vacuum break, which resulted in the layer sequence shown in Figure 2a. Secondly, an etch setup was used comprising a tailor-made PTFE holder without clamping to avoid any mechanical stress applied to the membrane in the configuration presented in Figure 2b. An etch duration of 90 min was found to reduce the effective RI repeatedly to 1.29. Note, the etch time had to be increased compared to the ‘porSiO_2_ on bulk’ approach because the temperature was intentionally set below the etchant’s boiling point of 158 °C (given by the supplier) to avoid membrane destruction. Besides the thickness reduction for the transformation from the as-deposited SiO_x_N_y_ to the porSiO_2_ layer by about 21%, the lowest layer of LPCVD SiN_y_ substrate membrane was simultaneously thinned by the etchant, which allowed for the controlled generation of ultra-thin membranes. Even though no reflux condenser was used, a linear etch rate of (0.65 ± 0.04) nm/min was found for the LPCVD SiN_y_ in hot H_3_PO_4_. However, the intermediate SiO_2_ layer is expected to remain unaffected since it was not directly accessible to the etchant and the etch rate is supposed to be even slower than in boiling H_3_PO_4_ (Figure 1b). Etch rates of 0.05 nm/min for SiO_2_ and of 0.20 nm/min for SiO_x_N_y_ were estimated from the total film thickness reduction for the 90 min etch duration. The remaining nanostructured porSiO_2_/SiO_2_/SiN_y_ membrane sketched in Figure 2c has an estimated thickness of 162 nm. Since the initial thickness of the deposited layers as well as the etch duration can be exactly adjusted, the synthesis route facilitates precise control of the final membrane’s thickness according to the needs. Note, the film thicknesses and the RIs of the multilayer structure on the free-standing membrane were not directly accessible by ellipsometry. Therefore, each layer was separately deposited on a bulk Si substrate. Then, ellipsometry measurements of the single layers were carried out before and after wet etching for 90 min in heated H_3_PO_4_. The individual fabrication steps of the membranes are summarized in Figure S1 in the Supplementary Materials.

### 3.3. Porous SiO_2_ Template—Catalyst-Assisted ZnO Nanowire Growth

**Figure 3:**
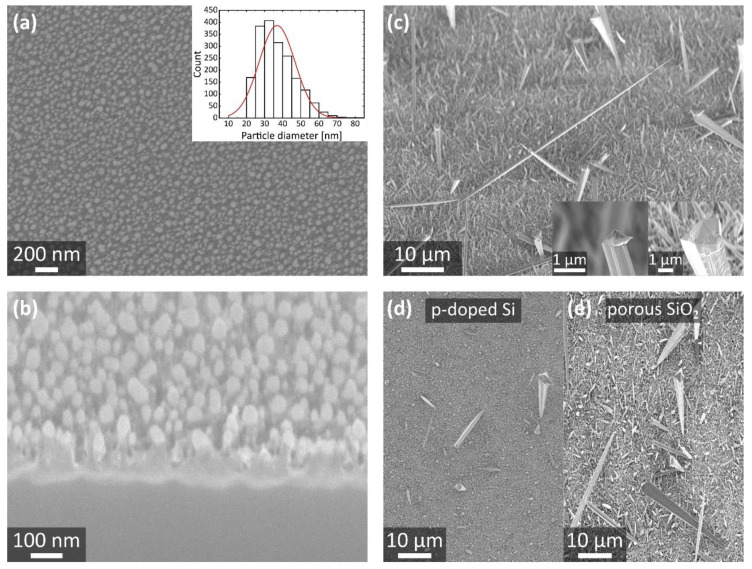
Thermally induced self-assembly of gold particles on porSiO_2_ and subsequent catalyst-assisted ZnO NW growth. (**a**) A mean particle diameter of (37 ± 10) nm was found for the densely packed gold dot distribution on the porSiO_2_ as presented by the histogram in the inset. (**b**) The gold particles were located on top of the porSiO_2_ rather than in the pores, which was revealed by the cross section through the substrate obtained by focused ion beam milling (30 kV, 50 pA). (**c**) The gold particles generated on the porSiO_2_ acted as catalysts for the subsequent growth of large, randomly oriented ZnO NWs by MOCVD. Compared to the result on the p-doped Si/native SiO_2_ substrate without porSiO_2_ (**d**), the ZnO NW growth yield was clearly enhanced on the porSiO_2_ layer (**e**). The SEM images in (**a**–**c**,**e**) were taken with the Crossbeam 550 by Zeiss. Note, (**b**) and (**c**) were taken at an angle of 54°. The SEM image in (**d**) was taken with the Supra 55 by Zeiss.

**Figure 4:**
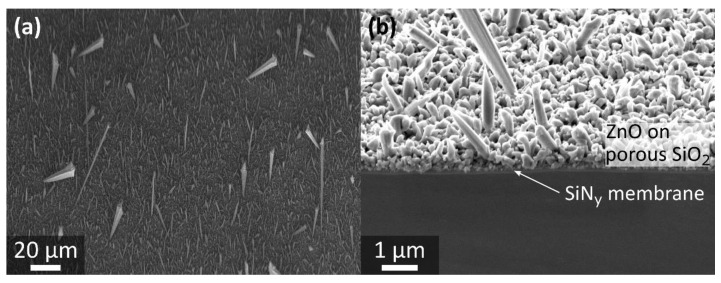
Catalyst-assisted ZnO NW growth on free-standing membranes. (**a**) Large ZnO nanowires, surrounded by shorter needles, were observed on the free-standing membranes. (**b**) The cross section through the free-standing membrane with porous SiO_2_ and ZnO structures on top was generated by focused ion beam milling (30 kV, 50 pA). The SEM images were taken at an angle of 54° with the Crossbeam 550 by Zeiss.

Metal particles are frequently employed as catalysts for the growth of nanostructures, such as ZnO NWs, to enhance and control their aspect ratios and to affect their density [13,29]. One option to distribute metal particles is by drop casting of a colloidal solution containing the (ideally monodisperse) catalyst particles, which is a simple and inexpensive method. However, this approach typically suffers from inhomogeneous particle distributions and particle aggregation, which would lead to generation of a non-uniform field emitter distribution. E-beam lithography in combination with physical vapor deposition or reactive ion etching is another preparation technique for metallic particles that comes with good control regarding the catalyst locations and sizes but turns out to be rather expensive and time-consuming [30]. An alternative method is the self-assembly of metallic particles by thermally induced solid-state dewetting of a thin film. When a textured substrate is used, the resulting particle pattern typically correlates with the surface structure of the underlying base, which was already shown for various porous substrates [12,30,31]. Herein, a thin gold film was deposited on the porSiO_2_ by sputter coating. After dewetting of the gold film on porSiO_2_ at a temperature of 580 °C, a mean particle diameter of (37 ± 10) nm was found (Figure 3a). As shown in the cross section through the sample in Figure 3b, the bright gold particles seem to sit on top of the porous structure rather than in the pores. Subsequently, the gold particles acted as catalysts for the ZnO nanostructure growth by MOCVD. Large ZnO NWs surrounded by shorter ZnO needles were observed on Si bulk samples (Figure 3c) as well as on the membrane substrates. A mean length and tip diameter of (49 ± 19) µm and of (0.110 ± 0.039) µm were found for the large ZnO NWs, respectively. Tapered wire ends with narrow tips as well as pyramidal structures with a base larger than the overall NW diameter were observed at the apexes of the large NWs, as exemplarily shown in the inset of Figure 3c. A similar ZnO NW morphology was observed on the free-standing membrane substrates, which is shown in Figure 4.

In general, a thinner metal film is needed to allow for the formation of smaller catalyst particles at the same dewetting temperature [12]. Note, the lower limit of the gold film thickness used herein (about (8.2 ± 0.4) nm) is defined by the minimum possible sputter time of the utilized sputter coater (K550X, Emitech) for a constant sputter current of 20 mA. The growth temperature has been fixed to the value that enables the formation of ZnO NWs in our growth setup (580 °C) [20]. Using these two constant parameters, only a few ZnO NWs were formed when an unstructured p-doped Si sample with its inherent native thermal SiO_2_ was employed as a substrate, as shown in Figure 3d. In contrast, a significantly enhanced ZnO NW growth yield can be noticed on the porSiO_2_ (Figure 3e), although the same initial gold film thickness was deposited on both samples. This observation is a strong indication that the porSiO_2_ positively affects the catalyst size distribution, possibly by a certain spatial confinement of the gold during particle agglomeration by dewetting. Elsewhere, it was observed that the particle diameter generally decreases for the same gold film thickness when a pre-patterned substrate instead of a plain one is used [32]. Note, smaller catalyst particles are expected to enhance the NW yield, which agrees well with our findings [33].

### 3.4. Porous SiO_2_ Template—Field Emission Measurements

**Figure 5:**
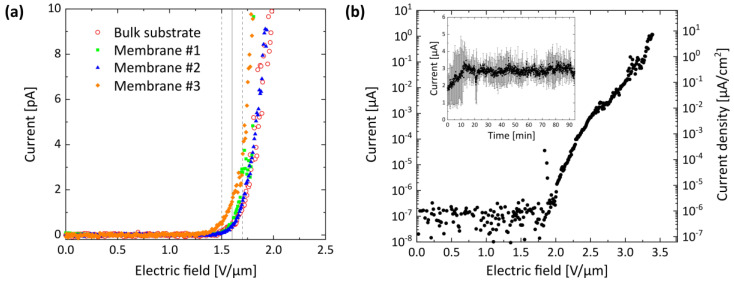
FE properties of ZnO NWs grown on free-standing membranes with porSiO_2_. (**a**) A mean onset field of (1.6 ± 0.1) V/µm was found for the three samples with ZnO NWs on membranes as well as for one ZnO emitter array on a Si bulk substrate indicating the high reproducibility of the ZnO growth process with the porSiO_2_. (**b**) Currents up to the microampere range were emitted from the ZnO NWs on a membrane substrate. The corresponding current density is displayed on the right axis, which reveals the turn-on field of 3.0 V/µm for a current density of 0.1 µA/cm^2^. Inset: The applied electric field of 3.4 V/µm was held constant two times for 94 min each, while the current was recorded to test the stability of the FE process. The emission current varied about 11% around a mean value of 2.8 µA. The error bars display the standard deviations of the data points, and only every fifth error bar is shown for clarity.

The FE properties of the ZnO NWs that were grown on porSiO_2_ generated with the gentler second etching method—using heated instead of boiling H_3_PO_4_ (Figure 2)—were investigated in two in-house built FE setups. To examine the reproducibility of the FE performance, the onset field was defined by the emission current overcoming a 10 σ threshold from the data mean with the standard deviation (σ) derived from the normal distribution of the measured current values. The onset fields of three ZnO NW-membrane samples grown on porSiO_2_ with an RI of about 1.29 varied only 10% around the mean of 1.6 V/µm (Figure 5a, filled symbols). For comparison, a ZnO NW array grown on a bulk substrate showed the same onset field (Figure 5a, empty circles). Note, Membrane #1 (plotted in green) was measured in the first FE setup (2 × 10^−4^ Pa) and the other samples were measured in the second FE setup (1 × 10^−5^ Pa). As displayed in Figure 5b, currents above 1 µA were obtained, but electrical discharge prevented further emission current rise. The turn-on field defined by an emission current density of 0.1 µA/cm^2^ was reached at an electric field of 3.0 V/µm (Figure 5b, right axis). Note, the emission current density was determined by division through the total macroscopic area that was exposed from the emitter array (0.16 cm^2^) [34]. Additionally, the stability of the emission current over time was tested as presented in the inset of Figure 5b since it is a critical property for the use as an FE-based sensor. The applied field was held constant at 3.4 V/µm, while the current was measured two times for 94 min each. An emission current of about 1.6 µA could be measured at the beginning, which increased to about (2.8 ± 0.3) µA within the first 10 min.

## 4. Discussion

The herein presented method for the fabrication of porSiO_2_ allows for the tailoring of the effective RI over a wide range from 1.46 (dense bulk-like SiO_2_) down to 1.20. The apparent porosity of the porSiO_2_ film was determined indirectly from the RI change generated by the selective wet etching. In general, the following two equations can be found in the literature to derive the apparent porosity using the RIs of the dense as-deposited layer ($n_{d}$), of the porous film ($n_{p}$), and of the surrounding medium, namely air ($n_{air}$ = 1.0) [2,4,9,35,36]:

(1) $$P\;\left[\%\right]=100\;\left(\frac{\left(n_{p\;}^{2}-n_{d\;}^{2}\right)\;\left(n_{air\;}^{2}+2n_{d\;}^{2}\right)}{\left(n_{p\;}^{2}+2n_{d\;}^{2}\right)\;\left(n_{air\;}^{2}-n_{d\;}^{2}\right)}\right)\;\mathrm{and}$$

(2) $$P\;\left[\%\right]=100\;\left(1-\frac{n_{p\;}^{2}-1}{n_{d\;}^{2}-1}\right).$$

However, both approaches are based on the effective medium theory that assumes the porous material as an optically isotropic medium that has an effective RI and that is constituted of two components, namely the carcass of a specific material and of the pores [2,37]. An apparent porosity range of 57 to 62% was derived for the RI reduction from 1.47 (SiO_x_N_y_) to 1.20 (porSiO_2_) with the two frequently used equations. Elsewhere, the porosity of SiO_2_ that was also prepared by a subtractive preparation route was reported to be about 55% for an effective RI of 1.20, which is in good agreement with our results [8]. Note, the porosity in their study was directly determined by the fitting of the measured ellipsometry data to the effective medium approximation model by adjusting the void percent.

For other porSiO_2_ synthesis methods, such as the sol-gel technique (1.14), a subtractive sequence of atomic layer deposition (ALD) and selective wet etching (1.13) or oblique angle deposition (1.05), RI values well below 1.20 have already been reported [8,38,39]. These extremely low RIs were not obtained in the present study, which is mainly attributed to the collapse of the porous structure caused by extended wet etching. However, the achieved RI would be sufficiently low for the porSiO_2_ to function as an ARC on glass substrates, as described in the introductory section [2]. Furthermore, the herein presented method avoids high annealing temperatures that are typically needed for the sol-gel technique [2,3]. Compared to the subtractive synthesis by ALD and subsequent wet etching, the deposition of the dense films by PECVD takes place at even lower temperatures (130 °C instead of 150 °C). Additionally, the synthesis time for porSiO_2_ is reduced by the higher deposition rates for PECVD ((22.5 ± 0.1) nm/min) compared to the ALD coating [8,9]. However, the herein presented synthesis method involves a wet etching step with an aggressive chemical at elevated temperatures. Thus, our method may not be applied to temperature-sensitive polymeric substrates. Other room-temperature deposition methods would be more suitable for the use of polymeric substrates, such as glancing angle deposition or the direct PECVD of porous SiO_2_ films [9,10,11,36,40]. The first approach that can be found in the literature for the generation of porous SiO_2_ solely by PECVD involves the deposition of a polymeric sacrificial layer followed by the deposition of a SiO_2_ film and the simultaneous removal of the polymer, which leads to the formation of a porous structure [10]. For the second method, the porous films are generated by the accumulation of particles that condensed already in the gas phase of the PECVD process, which is accomplished by precise tuning of the deposition parameters, such as pressure, temperature, gas flow ratios, and by modification of the deposition reactor geometry [9,11,40]. Typically, specific reactor designs were used, such as a PECVD equipped with an electron cyclotron resonance microwave reactor or a remote PECVD, which allows for the physical separation of the region of RF discharge from the area of the substrate [10,11,41]. The maximum porosity values achieved with the two direct PECVD methods vary in a range of 41% up to 65%, which is comparable to our result [9,10,41].

Investigation of the thin films at different stages of the synthesis process may allow to examine the evolution of the etching process in the material, which could indicate methods for a stronger RI reduction before the collapse of the porSiO_2_ occurs. Possibly, X-ray photoelectron spectroscopy could be used to study the chemical composition of the thin films at various phases of their synthesis to supplement the findings of the ellipsometry measurements [9]. Furthermore, high-resolution transmission electron microscopy may be used to investigate the pore development through the thin films [7]. Additionally, ellipsometric porosimetry could be an option to determine the pore size and distribution of our porSiO_2_ films in the future [41,42].

ZnO NWs were successfully grown on free-standing membranes with porSiO_2_ and employed as FE electron sources, which demonstrates the durability of the samples at elevated temperatures as well as their mechanical strength. By application of a catalyst, the generated ZnO NWs are about 10 times larger than the ZnO structures that were fabricated in our previous work by a similar, but catalyst-free, MOCVD process [20]. Consequently, the onset field was reduced from about 30 V/µm (previous work) to only 1.6 V/µm through the enhanced emitter aspect ratio. The extracted turn-on field of 3.0 V/µm (for 0.1 µA/cm^2^) is comparable to the values given elsewhere to describe the performance of ZnO field emitters that were also grown by vapor phase methods but with other precursor substances on bulk substrates, namely 0.85 V/µm up to 6.0 V/µm [43]. Note, the observed increase of the steady-state current that appeared within the first 10 min of the measurement may be attributed to the removal of foreign species from the emitter by the local temperature increase caused by resistive self-heating in combination with the inhibited re-adsorption on the heated surface [44,45].

## 5. Conclusions

We present a low-temperature method for the synthesis of porSiO_2_ on bulk as well as on free-standing membrane substrates. A minimum effective RI of 1.20 was obtained, which corresponds to an apparent porosity of roughly 60%. The complete processing route remains below temperatures of 160 °C, and the method would principally be capable of high throughput because of the fast PECVD deposition rates. A future detailed structural investigation of the pore formation process by means of transmission electron microscopy may allow to precisely determine the timing for the collapse of the porSiO_2_. The nanostructured porSiO_2_ surface was successfully employed as a substrate for the growth of ZnO field emitters, which indicates the mechanical strength and temperature stability of the samples. The emitters on the free-standing membranes may pave the way for future FE-based sensor devices, such as mass spectrometric detection systems, because of their convenient FE performance [19]. Additionally, the synthesis route for the fabrication of porous layers on free-standing membranes could be used for modification of commercial diffusion membranes [10]. Moreover, the as-deposited SiO_x_N_y_ films may be investigated further since their optical, electrical, and also mechanical properties can be tuned over a wide range by adjusting their RI between the values of pure SiO_2_ (1.46) and of SiN_y_ (2.1). SiO_x_N_y_ is known for its enhanced resistance to specific chemicals and to impurity diffusion, which makes it a promising candidate for the replacement of pure SiO_2_ in microelectronic devices or as a scratch resistant coating on transparent polymeric substrates for optoelectronic applications [46,47].

## Data Availability

Data are contained within this article. Further data of this study are available from the corresponding author upon reasonable request.

## Supplementary Materials

The following are available online at https://www.mdpi.com/article/10.3390/nano11123357/s1, Figure S1: Synthesis steps for the porous SiO_2_ on a free-standing membrane.
