# Supplementary material for: Subtractive Low-Temperature Preparation Route for Porous SiO2 Used for the Catalyst-Assisted Growth of ZnO Field Emitters
Source: Nanomaterials (Basel). 2021 Dec 10;11(12):3357. doi: 10.3390/nano11123357 (PMC8709353; doi:10.3390/nano11123357)
Supplement: Supplementary file 1 [file nanomaterials-11-03357-s001.zip › nanomaterials-1460115-supplementary.pdf]

Supplementary Materials:

Subtractive Low-Temperature Preparation Route for Porous SiO_2 Used for the Catalyst-Assisted Growth of ZnO Field Emitters

Stefanie Haugg [1], Carina Hedrich [1], Robert H. Blick [1,2] and Robert Zierold [1,*]

[1] Center for Hybrid Nanostructures (CHyN), Universität Hamburg, 22761 Hamburg, Germany; shaugg@physnet.uni-hamburg.de (S.H.); chedrich@physnet.uni-hamburg.de (C.H.); rblick@physnet.uni-hamburg.de (R.H.B.)
[2] Material Science and Engineering, College of Engineering, University of Wisconsin-Madison, Madison, Wisconsin 53706, USA
* Correspondence: rzierold@physik.uni-hamburg.de

Synthesis of Porous SiO_2 on Free-Standing Membranes

For the synthesis of the porous SiO_2 film on a free-standing membrane, first a SiN_y membrane substrate was fabricated from a commercially bought Si wafer that is covered on both sides with LPCVD SiN_y. Second, the free-standing SiN_y membrane was coated by PECVD with a layer of SiO_2, followed by a layer of SiO_xN_y. Third, the $SiO_xN_y/SiO_2/SiN_y$ membrane is etched for 90 min in heated H_3PO_4, which leads to the generation of a porous SiO_2 layer. Thereby, the effective RI is reduced from 1.474 (SiO_xN_y) to 1.290 (porous SiO_2).

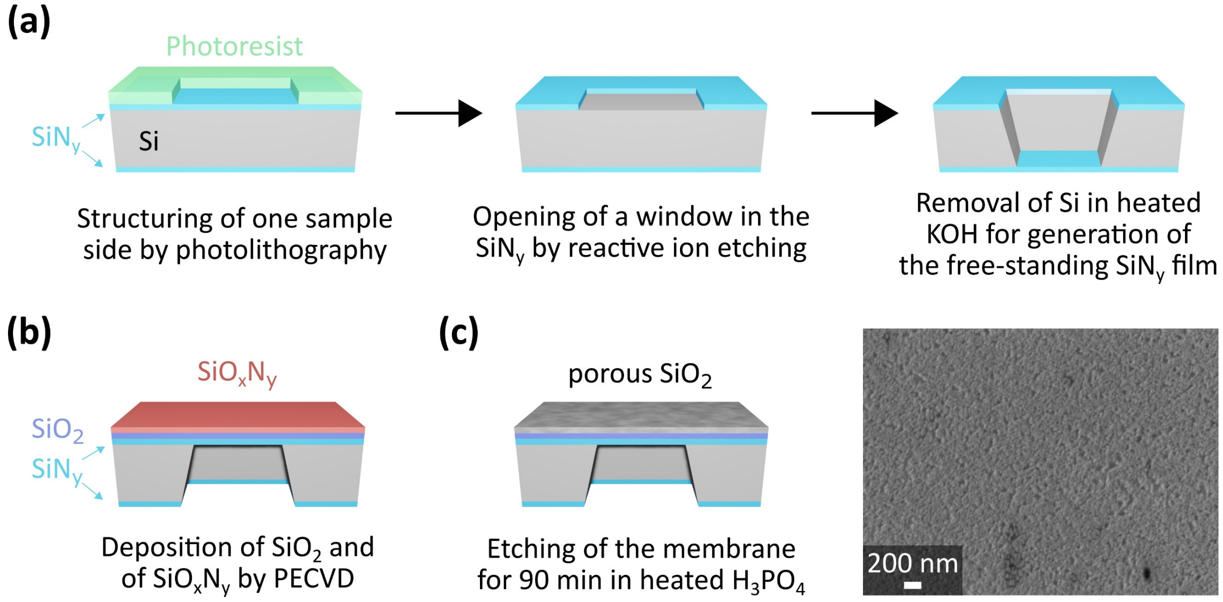

Figure S1: Synthesis steps for the porous SiO_2 on a free-standing membrane. (**a**) The SiN_y membrane was fabricated from a Si wafer with LPCVD SiN_y on both sides. After a mask was defined on one side of the sample by photolithography, a window was opened in the SiN_y film by reactive ion etching. Then, the heated KOH (30 %, 80 °C) was used to remove the Si substrate, which led to the generation of a free-standing SiN_y film. (**b**) SiO_2 and SiO_xN_y were successively deposited by PECVD without a vacuum break. (**c**) A porous SiO_2 layer was generated on the surface of the membrane by wet etching in heated H_3PO_4. The SEM image of the porous layer on a membrane was taken with the Crossbeam 550 by Zeiss.
